# Supplementary material for: Internal thread defect detection system based on multi-vision
Source: PLoS One. 2024 May 28;19(5):e0304224. doi: 10.1371/journal.pone.0304224 (PMC11132497; doi:10.1371/journal.pone.0304224)
Supplement: Attachment — Submitted filename: Response to reviewer.pdf [file pone.0304224.s002.pdf]

We sincerely appreciate the valuable comments and suggestions from the editors and anonymous reviewers, which have significantly improved the quality and presentation of our manuscript. Below, we will detail how we integrated these feedbacks and the reviewers' suggestions during the revision process.

Journal Requirements

1. Please ensure that your manuscript meets PLOS ONE's style requirements, including those for file naming.

Author response: Thank you for your guidance and for highlighting the importance of adhering to PLOS ONE's style and file naming requirements. I assure you that we have carefully reviewed the journal's guidelines to ensure full compliance.

2. Please note that PLOS ONE has specific guidelines on code sharing for submissions in which author-generated code underpins the findings in the manuscript.

Author response: Thank you for the reminder of PLOS ONE's code sharing policy. I understand the importance of this policy in ensuring transparency and reproducibility of research results. I respect the journal's commitment to open science standards. However, due to personal reasons and constraints, I am currently unable to upload all of the code, but I can provide the key defect detection code. Thank you for your understanding and consideration of our special circumstances in this submission. I look forward to your guidance on how to proceed.

3. Please confirm at this time whether or not your submission contains all raw data required to replicate the results of your study. Authors must share the “minimal data set” for their submission. PLOS defines the minimal data set to consist of the data required to replicate all study findings reported in the article, as well as related metadata and methods.

Author response: Thank you for your guidance. Given that our study utilized only a subset of data, we will be uploading this subset as the minimal data set, in compliance with PLOS's requirements for the minimal data set.

Review Comments

Reviewer #1:

1. The contributions of this study should be proposed clearly.
2. The advantages of the proposed approach should be compared to other current approaches, including Image Preprocessing, Image Stitching, especially Defect Detection.
3. Some quantitative results should be proposed in the conclusion section.

Author response: We express our profound gratitude for the considerable time and

meticulous attention you have dedicated to the review of our manuscript. It is with great appreciation that we acknowledge your recognition of our research efforts.. We have modified the introduction and discussion sections to more accurately elucidate the contributions of our research. We now clearly outline the theoretical and practical significance of our findings, as well as their relevance within the wider scope of research. We have enriched the content of our paper by directly contrasting the proposed method with existing approaches in Image Stitching and Defect Detection. This comparison highlights the superior accuracy, efficiency, and computational cost-effectiveness of our method compared to current technologies. The conclusion section has been revised to summarize the quantitative results that demonstrate the efficacy of our approach.

Reviewer #2:

This manuscript proposes a vision-based approach that significantly enhances the efficiency of image acquisition through sophisticated hardware architecture and addresses particular challenges in industrial inspection, contributing to the field of internal thread defect detection. However, the paper is deficient in innovation, and there is a notable absence of requisite experimental results and analytical discussion. The author should provide additional experimental data to delineate the methodology's advantages and innovations in comparison to alternative techniques for internal thread defect detection.

Author response: We greatly appreciate the time and effort you have dedicated to reviewing our manuscript, and we thank you for acknowledging the potential of our vision-based approach in the field of internal thread defect detection. We have carefully revisited the literature review to better accentuate the innovative aspects of our hardware architecture and vision-based method. Following your suggestion, we have compiled a more comprehensive set of experimental results, which not only affirm the efficiency and effectiveness of our approach but also demonstrate its advantages over existing technologies. We have expanded the discussion section to include a more in-depth analysis of the experimental outcomes. This involves a critical comparison with other techniques, providing unequivocal evidence of the strengths and potential areas for improvement of our method. We trust that these enhancements will address the issues you have raised and are hopeful that the revised manuscript will meet the esteemed standards of your publication. We are dedicated to making a substantial contribution to the field and eagerly anticipate the opportunity for our work to be featured in your journal.we have highlighted the responses for each query in the manuscript annotated next to the manuscript. Besides, we integrated the comments of the reviewers and gave detailed answers to each comment, clearly marking the position of the modification. As follows:

1.In the abstract, the authors are encouraged to include empirical data to delineate the superiority of their approach over manual inspection or other existing methods for detecting internal thread defects, as this would lend greater persuasive power to their claims.

Author response: Thank you for your insightful suggestion regarding the enhancement of our manuscript’s abstract. We agree that the inclusion of empirical data within the abstract would significantly strengthen the presentation of our research by quantitatively demonstrating the superiority of our approach.To this end, we have now incorporated key empirical findings that clearly exhibit the advantages of our method over manual inspection and other existing techniques in detecting internal thread defects. We believe that this addition will provide readers with a concise yet compelling evidence of our approach's efficacy, right at the outset of the paper.

The specific location is as follows: Lines 8 to 20

In the realm of industrial inspection, the precise assessment of internal thread quality is crucial for ensuring mechanical integrity and safety. However, challenges such as limited internal space, inadequate lighting, and complex geometry significantly hinder high-precision inspection. In this study, we propose an innovative automated internal thread detection scheme based on machine vision, aimed at addressing the time-consuming and inefficient issues of traditional manual inspection methods. Compared with other existing technologies, this research significantly improves the speed of internal thread image acquisition through the optimization of lighting and image capturing devices. To effectively tackle the challenge of image stitching for complex thread textures, an internal thread image stitching technique based on a cylindrical model is proposed, generating a full-view thread image. The use of the YOLOv8 model for precise defect localization in threads enhances the accuracy and efficiency of detection. This system provides an efficient and intuitive artificial intelligence solution for detecting surface defects on geometric bodies in confined spaces.

2. Within the introduction, several alternative methods for internal thread defect detection are mentioned, yet there is a lack of detailed discussion regarding their limitations. To better establish the proposed method's advancement, these shortcomings should be elucidated.

Author response: In response to your valuable suggestion, we have revised the introduction to include a more detailed examination of the inherent limitations of current methods used for detecting internal thread defects. This elucidation covers aspects such as accuracy, efficiency, and applicability to different materials among these alternative approaches. By contrasting these limitations with the advantages of our proposed method, we aim to highlight the contributions our research makes to the field.

The specific location is as follows: Lines 56 to 64, Lines 76 to 92

Lines 56 to 64:

In recent years, research on external thread detection has advanced considerably. Detection methods can be broadly classified into two categories: contact and non-contact. Contact methods encompass techniques such as thread gauge detection (qualitative), measuring needle detection (including two-needle and three-needle methods), thread micrometer inspection (single inspection), sample inspection, three-coordinate measurement [1], and contact scanning. Although these methods tend to be more accurate, they require skilled inspectors using specialized tools for precise operation, leading to higher labor costs and lower efficiency. Additionally, they are susceptible to subjective bias and may cause wear on the workpiece during inspection. In contrast, non-contact methods utilize optical principles, including techniques like optical microscope inspection [2], laser scanning [3], and machine vision [4]. These methods simplify certain manual tasks and complex calculations through high-precision inspection equipment, enabling automated control and enhanced accuracy. Machine vision-based defect inspection, leveraging images captured by specific light sources and hardware, significantly improves the quality, efficiency, and reliability of defect detection. However, due to the influence of the diffraction limit, optical microscopes have inherent limitations in resolution, and the depth of field of optical microscopes is usually shallow. For thicker samples, it is impossible to keep the entire volume in a clear focal plane simultaneously. For sensitive materials, the use of strong lasers may cause surface damage or changes to the sample, thereby affecting the accuracy of detection results. Compared with the previous two defect detection methods, the defect detection method based on machine vision can quickly process and analyze images, achieving high-speed automatic detection, significantly improving the detection speed and efficiency of the production line, and is suitable for various types and complexities of defect detection tasks.

Lines 76 to 92:

For defect detection of internal threads, high-precision laser sensors [5] can be used. Through the integration of high-precision optical detection and ranging (LiDAR) technology and cameras [6], accurate detection of structural cracks has been achieved. This method can be applied to the detection of defects in internal threads to verify their quality. However, this method does not perform well on reflective surfaces or transparent materials, as the laser might be scattered or refracted, affecting the accuracy of measurements. Image-based systems, utilizing linear lasers and template matching techniques [7], can quickly detect thread defects. Yet, for parts with

complex or non-standard thread shapes, it may be necessary to create multiple templates, increasing the system's complexity and computational demands. Utilizing novel arrayed differential flexible eddy current sensors [8], defects on the surface of iron threads can be detected, but the detection effectiveness for non-metallic materials is limited, and the performance of eddy current sensors may be influenced by the conductivity of the test materials. Through digital image processing techniques [9], OTPG can standardize and segment internal thread images, further assessing their quality. This method depends on the quality of images, and the accuracy of the processing algorithms decreases in conditions of insufficient light or poor image quality. Moreover, complex image processing algorithms require higher computational resources and processing time, affecting the real-time nature of detection.

3. The authors may add more state-of-art computer vision articles for the integrity of the manuscript (Real-Time Defect Detection for Metal Components: A Fusion of Enhanced Canny-Devernay and YOLOv6 Algorithms; Applied Sciences. 3D vision technologies for a self-developed structural external crack damage recognition robot; Automation in Construction.).

Author response: Thank you for your suggestion Following your valuable recommendation, we have added the literature you suggested, thereby enhancing the content of our article.

The specific location is as follows: Lines 77 to 78,Lines 99 to 104

Lines 77 to 78:

Through the integration of high-precision optical detection and ranging (LiDAR) technology and cameras [6], accurate detection of structural cracks has been achieved.

Lines 99 to 104:

While the aforementioned methods enable the detection of internal thread defects, they are not without their limitations. The current state of research in machine vision, recognized for its efficacy in defect detection, is as follows: Machine vision has achieved high accuracy and precision in detecting weld defects in thin-walled metal cans [10]; it is effectively employed in the detection of traps in printed circuit boards [11], an approach that is independent of color, position, and direction, enabling rapid and accurate defect identification; similarly, machine vision is extensively utilized for detecting surface defects in iron and steel products [12]. Real-time defect detection of metal components [13] has significantly enhanced the accuracy and speed of identifying surface flaws and measuring dimensions in metal parts by employing

YOLOv6 and an improved Canny–Devernay subpixel edge detection method. For internal thread defect detection, machine vision, compared to other methods, maintains detection speed while ensuring the accuracy of the detection results.

4. The YOLOv8 model, as trained by the authors for the detection of internal thread defects, must have its training outcomes displayed, including key performance metrics such as recall and precision rates.

Author response: Thank you for your valuable feedback. We have incorporated the training results of YOLOv8, presenting key metrics such as recall rate, accuracy, and mAP, and compared these with the training outcomes of SSD and YOLOv5. This comparison underscores the superiority of YOLOv8 in detecting internal thread defects.

The specific location is as follows: Lines 459 to 465

The Table 3 below compares the training parameters of YOLO v8, YOLOv5, and SSD, quantifying the training outcomes of these three algorithms based on mAP, recall, and precision. Due to limitations in detecting small targets or specific types of defects, SSD has a lower recall rate. YOLOv5 and YOLOv8 show stronger performance in detecting small targets, with YOLOv8 outperforming in terms of accuracy and recall rate, exhibiting superior parameters compared to both SSD and YOLOv5.

TABLE 3

Comparison of training parameters for YOLO v8, YOLOv5, and SSD

Feature	mAP	recall	precision
SSD	71.10%	46.67%	87.50%
YOLOv5	91.47%	73.33%	86.23%
YOLOv8	91.56%	84.68%	88.36%

5. Figure 17 lacks clarity. It is recommended that the authors re-upload this image with improved resolution or clarity.

Author response: Thank you for your guidance.We have replaced it with images of higher clarity.

6. The experimental section is wanting in specific data related to internal thread defect identification, such as the count and percentage of detected defects.

Author response: Thank you for your valuable suggestions. We have re-uploaded and organized the data on internal thread defect detection, providing details on the types of defects and their percentages.

The specific location is as follows: Lines 467 to 472

In the detection of collected thread images, as shown in Figure 16, defects on the internal threads are marked with red boxes. This project has designated these defects as "corrosion," with the latter being the probability of identifying them as defects, achieving precise detection. The figure below randomly inspects 8 internal thread images (A, B, C, D, E, F, G, H), with a total of 19 defects. Among these, defects in 6 images were completely detected, identifying 17 correct defects in total. The sample average detection rate is 89.47%.

7. The inclusion of this data is recommended.The conclusion of the manuscript should synthesize the experimental findings to provide a cohesive summary of the research's implications and results.

Author response: In response to your feedback, we have revised the conclusion of our manuscript to ensure it synthesizes the experimental findings effectively. We now provide a cohesive summary that articulates the research's implications and results, thereby offering a clearer understanding of the study's significance and contributions to the field. Thank you for guiding us to enhance the clarity and impact of our work.

The specific location is as follows: Lines 518 to 526

This article addresses the challenges related to internal thread detection and proposes an automated detection solution based on machine vision, with the following main contributions:

1、 The article designs an efficient image acquisition system for internal thread defect detection by improving lighting conditions and image capture devices, enhancing the quality of internal thread surface images and greatly increasing the efficiency of image acquisition.

2、 A method based on a cylinder model for stitching internal thread images is proposed to provide full-field thread images, solving the challenge of stitching such complex textured images.

3、 Utilizing the YOLOv8 model, the system accurately locates the positions and sizes of thread defects, demonstrating the effectiveness of this system solution.
