# Supplementary material for: Internal thread defect detection system based on multi-vision
Source: PLoS One. 2024 May 28;19(5):e0304224. doi: 10.1371/journal.pone.0304224 (PMC11132497; doi:10.1371/journal.pone.0304224)
Supplement: S1 File — (ZIP) [file pone.0304224.s001.zip › ultralytics-main/docs/overrides/main.html]

{% extends "base.html" %}
{% block announce %}

Introducing Ultralytics HUB Cloud Training! ☁️ Scalable. Simple. Smart.   ➜

{% endblock %}
