# Supplementary material for: Internal thread defect detection system based on multi-vision
Source: PLoS One. 2024 May 28;19(5):e0304224. doi: 10.1371/journal.pone.0304224 (PMC11132497; doi:10.1371/journal.pone.0304224)
Supplement: S1 File — (ZIP) [file pone.0304224.s001.zip › ultralytics-main/docs/overrides/partials/comments.html]

{% if page.meta.comments %}

## {{ lang.t("meta.comments") }}


{% endif %}
